# Multiple antibiotic resistance indexing and molecular identification of *Escherichia coli* isolated from clinical and nonclinical sources in Port Harcourt Metropolis, Nigeria

**DOI:** 10.11604/pamj.2025.51.11.38524

**Published:** 2025-05-13

**Authors:** Doubra Otis Isaiah, Kome Otokunefor, Obakpororo Ejiro Agbagwa

**Affiliations:** 1 Department of Microbiology, Faculty of Science, Niger Delta University, Bayelsa State, Nigeria,; 2 Department of Microbiology, Faculty of Science, University of Port Harcourt, Rivers State, Nigeria

**Keywords:** Antibiotic resistance, molecular identification, *Escherichia coli*, Port Harcourt, Nigeria

## Abstract

**Introduction:**

the emergence of antibiotic resistance in E. coli strains has led to a variety of clinical illnesses in both humans and animals, with consequences for human health and the environment.

**Methods:**

this cross-sectional study was conducted to determine the risk of increased antibiotic resistance by calculating the multiple antibiotic resistance (MAR) index of E. coli in Port Harcourt, Rivers State, Nigeria. A higher MAR index close to 1 was interpreted as having a higher level of antibiotic resistance, while a lower MAR index close to 0 is an indicative of little to no resistance. In this study, a total of 200 samples from clinical (urine and stool) and non-clinical sources (soil and poultry) were randomly collected following ethical approval. Escherichia coli was isolated on Eosin Methylene Blue agar and identified using standard biochemical tests. The antimicrobial susceptibility profile was determined using the disk diffusion methodology with Mueller-Hinton agar according to the instructions of the Clinical and Laboratory Standards Institute (CLSI). Escherichia coli isolates were amplified in this investigation using a 16s rRNA gene-based polymerase chain reaction (PCR) technique.

**Results:**

for both 73% clinical (n=100) and 35% non-clinical (n=100) isolates, amoxicillin/clavulanic acid showed the highest rate of resistance with 82.2% (60 of 73) and 100% (35 of 35) respectively, whereas nitrofurantoin showed the lowest rate of resistance at 1.4% (1 of 73) clinical and no resistance was found for the nonclinical E. coli isolates. The study presumptively finds a higher resistance to 4 drug classes, with 42%, and the lowest of 1% to 6 drug classes of antibiotics. Additionally, MAR indices > 0.2 were observed in this study, which indicates excessive use of antibiotics. MAR index of 0.4 was the most frequent, with a 25% prevalence. The results of this investigation show that a MAR index of 0.4 suggests widespread antibiotic resistance, with 25% of the bacterial isolates from both sources exhibiting resistance to the tested antibiotics. A MAR index > 0.2 in this study indicates that 14.8% of the E. coli isolates were ineffective against the tested antibiotics. The bacteria isolates in this study have developed resistance to multiple antibiotics if the MAR index is greater than 0.2, which is an indication of overuse or inappropriate antibiotic use. The genotypic test verified 82.9% non-clinical E. coli (found in environmental soil and poultry samples) and 82.2% clinical E. coli (found in patient urine and stool samples). In this study, E. coli isolates were genotypically identified using the 16s rRNA gene.

**Conclusion:**

the high levels of MAR indices in E. coli as presented in this study could be indicative of antibiotic treatment failures in both clinical and nonclinical settings, which can serve as a potential reservoir of drug-resistant E. coli that are harmful and therefore require continuous monitoring.

## Introduction

The growing use of antibiotics in humans and animals has raised several concerns regarding the health of both. The primary source of concern has been the phenotypic proliferation of antibiotic resistance in the typical commensal microbiome as well as in clinically important strains [1]. Since antimicrobial drugs have been used to treat illnesses for so long, different bacterial strains have developed resistance to them, which has led to poor treatment and infection spread [2,3].

*Escherichia coli* can cause a variety of diseases in humans and animals, and it´s a model for examining antimicrobial drug resistance. When bacteria become resistant to many drugs, antibiotics lose their ability to treat and control illness. The Multiple Antibiotic Resistance (MAR) index is used to assess the risk of *E. coli* in studies, indicating areas with high antibiotic abuse rates. It helps understand the broader impacts of antibiotic resistance spread. Bacteria with a high MAR index are likely from environments like soil, hospitals, or water. The environment contains more of these resistance genes [4].

Antimicrobial resistance in *E. coli* is a serious hazard to public health and is occurring at an alarming rate worldwide. Antibiotic resistance genes are widely distributed in clinical and environmental sources, and a significant problem that has not yet been resolved is the potential for resistant genes to be transferred to humans and animals through interaction with different environmental sources, as well as to non-resistant strains of organisms [5]. According to studies, unsafe environments are responsible for 80% of all illnesses [6].

The World Health Organization states that antibiotic resistance is a global emergency and causes approximately 700,000 deaths globally. This is because microorganisms have developed resistance to antimicrobial drugs, making them ineffective in treating and managing diseases [7-9]. Multidrug-resistant *E. coli* strains with an exceptionally high MAR index rate of ≥ 0.8 have been reported in a few studies from this area. Since the MAR index rate in human, animal, and environmental samples indicated unsuccessful antibiotic treatments and could be a source of potentially dangerous drug-resistant organisms, it was necessary to continuously monitor it [1,3]. Nigeria and the rest of the globe have yet to find a complete solution to the growing global danger of antibiotic resistance. The current study aimed to determine the MAR index of *Escherichia coli* isolated from clinical and non-clinical sources in Rivers State, as well as the prevalence of *E. coli*.

## Methods

**Study design and settings:** clinical and non-clinical samples were analyzed at random using a cross-sectional study that was based in a hospital and an environment. *Escherichia coli* isolated from clinical and non-clinical sources is molecularly identified in this study using 16S rRNA genes specific for *E. coli* (EC16 primer pairs specific for *E. coli*). The study was carried out in South-South Nigeria's Rivers State, specifically in Port Harcourt, which is the state capital.

**Study population:** the research population consists of both in-patients and out-patients receiving care at the University of Port Harcourt Teaching Hospital. While refusal of individuals is an exclusion criterion, both in-patients and out-patients who consented to have their samples used for this study are regarded as inclusion criteria. All ages and genders are included in this study; it is not gender-exclusive.

### Laboratory analysis

**Sample processing and *Escherichia coli* isolation and characterization:** for this analysis, 200 samples in all, equally split between clinical and non-clinical sources, were used. Isolates were extracted from soil, poultry, urine, and stool. While stool samples were combined with normal saline before being inoculated onto Eosin Methylene Blue Agar (EMB), clinical urine samples were inoculated directly. Before inoculating EMB using the spread plate method, the soil samples and chicken droppings were serially diluted. The samples were then incubated for 24 hours at 37°C. To obtain a distinct colony for additional biochemical identification, such as Indole, Methyl-red, Voges Proskauer, and Citrate Utilization test (IMViC test), the isolates were further subcultured [10].

**Phenotypic detection using a drug susceptibility test disc:** the Kirby Bauer disc diffusion technique [11], which entails inoculating a 0.5 McFarland concentration onto the surface of a sterile Mueller Hinton agar plate [10], was used to determine the resistance profile of *Escherichia coli* isolates. The process entails standardizing the bacterial isolates to a turbidity standard equal to 0.5 McFarland. Before inserting the commercially available antibiotic-impregnated filter-paper discs using sterile forceps, the surface of freshly prepared Mueller-Hinton Agar was thoroughly swabbed with the bacterial isolate. Following a 24-hour incubation period at 37°C, the zone of inhibition was measured to the closest millimeter and classified as susceptible, intermediately sensitive, and resistant [12].

**Multiple Antibiotic Resistance (MAR) index:** the a/b formula [13] was used to determine the multiple antibiotic resistance index (MAR index) for each *E. coli* isolate, where “a” is the total number of antibiotics to which the isolated organism is resistant and “b” is the total number of test antibiotics that the isolated organisms were tested for. Only nine antibiotics were tested against the isolated organism in this investigation. The antimicrobial susceptibility test results were used to calculate the MAR index. A high-risk source of contamination where antibiotics are primarily used is indicated by a MAR index value greater than 0.2, whereas a source associated with low antibiotic use is indicated by a MAR index value less than 0.2 [14]. The results of the antibiotic susceptibility testing were also used to determine the isolates' antibiotic profile and multidrug resistance.

**DNA extraction and molecular characterization of *Escherichia coli* isolates:** as directed by the manufacturer (Geneaid Biotech, Ltd., Taiwan), *Escherichia coli* DNA was extracted using the PrestoTM Mini gDNA Bacteria Kit Quick Protocol. The Nano-Drop Spectrophotometer (nanodrop ND 1000), which has a purity range of 1.8 to 2.0, was used to measure the concentration and purity of the extracted DNA [15]. Using *E. coli*-specific 16s rRNA gene fragment of Ec16 primer pairs, *Escherichia coli* isolates were further verified (Table 1). 3µl of *E. coli* DNA, 10µl PCR master mix, 1µl of each of the two primers, and 6µl of nuclease-free water were added to create the reaction mixture [16].

**Table 1:** primer sequence for the molecular identification of *Escherichia coli* isolates

Gene	Primer name	Primer sequence (5' to 3')	Annealing temp (°C)	Product size (bp)
EC16	EC16 F	5’-GACCTCGGTTAGTTCACAGA-3’	55°C	588bp
	EC16 R	5’-CACACGCTGACGCTGACCA-3’		

**Data collection:** to document MAR indices, antibiotic resistance profiles, and molecular characterization results, data sheets were made. A prominent band at 588bp on the agarose gel indicated a positive PCR result for the identification of *E. coli*. The zone of inhibition was measured using a meter rule, and the results were compared with the standard set by the CLSI standards.

**Definitions:** MAR index: bacterial isolates' level of antibiotic resistance is determined by the MAR Index, which is based on the ratio of antibiotics resistant to total antibiotics (formula a/b).

*Escherichia coli*: both human and animal intestines are commonly home to the rod-shaped, Gram-negative bacterium *Escherichia coli* (*E. coli*), which is known to cause a variety of diseases.

Molecular identification: this is the process of employing genetic techniques (like PCR) to identify and characterize bacterial isolates. Antimicrobial Resistance: this, or AMR, is the capacity of microorganisms to resist the actions of antimicrobial agents.

**Statistical analysis:** using Microsoft Excel version 2021, averages, means, and percentages were computed. Frequency tables and charts were produced when the data was analyzed using the proper statistical technique. Patterns of resistance to multiple antibiotics were used to calculate each isolate's MAR index.

**Ethical consideration:** before the study started, the ethical committees of the University of Port Harcourt and the University of Port Harcourt Teaching Hospital (UPTH) were consulted for approval (UPTH/ADM/90/S.11/VOL.XI/1110).

## Results

**Figure 1:**
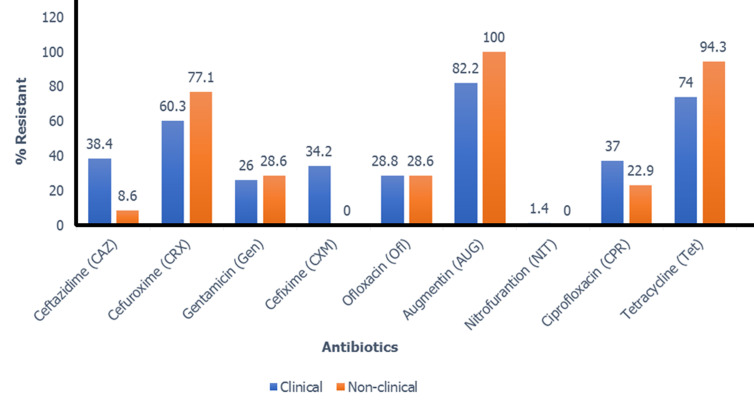
comparison of antibiotic resistance among *Escherichia coli* isolates from clinical and nonclinical sources

A total of 73 clinical and 35 non-clinical *E. coli* isolates were identified biochemically in this investigation, which involved 200 samples. Additionally, 60 clinical and 29 non-clinical isolates were confirmed using genotypic techniques. The proportion of occurrence indicates that *Escherichia coli* was distributed differently in each of the tested samples. Clinical samples had the highest concentrations of *Escherichia coli* (73%; 73 of 100) compared to non-clinical samples (35%; 35 of 100). According to a study on antimicrobial susceptibility, clinical isolates have a higher rate of resistance than non-clinical isolates (Figure 1). In both clinical and non-clinical isolates, amoxicillin/clavulanic acid had the highest resistance rate (100% and 82.2%, respectively), while nitrofurantoin had the lowest.

Based on an analysis of the antibiotic profile shown in Table 2, 43 antibiotic profiles were discovered among the *E. coli* strains recovered for this study. Stool, soil, poultry, and urine all have distinct antibiotic profiles, which are 22, 28, 9, and 9 in that order. In all four of the sample types examined in this study, none of the profiles were found. The most frequently found profile in 11 *E. coli* isolates was CRX-AUG-TET, which was followed by CRX-GEN-AUG-TET in 10 isolates.

**Table 2:** antibiotic profile and multidrug resistance pattern of antibiotic-resistant *E. coli* isolates

		Clinical samples		Non-clinical			
S/No	Antibiotic profile	Urine	Stool	Soil	Poultry	No. of drug classes	MDR
1	AUG	2	1	3	-	1	-
2	TET	1	1	-	-	1	-
3	AUG-TET	1	5	1	-	2	-
4	CRX-AUG	3	-	1	2	2	-
5	CAZ-CRX-CXM-TET	1	-	-	-	2	-
6	CAZ-CRX-CXM-AUG	-	2	-	-	2	-
7	CAZ-CRX-CXM-TET	-	1	-	-	2	-
8	CRX-AUG-TET	5	-	3	3	3	+
9	CAZ-AUG-TET	1	1	-	-	3	+
10	GEN-AUG-TET	2	-	-	-	3	+
11	AUG-CPR-TET	1	3	-	-	3	+
12	CXM-AUG-TET	-	2	-	-	3	+
13	OFL-AUG-TET	-	-	1	-	3	+
14	CRX-OFL-CPR-AUG-TET	2	1	-	1	4	+
15	CRX-OFL-CPR-AUG-NIT-TET	1	-	-	-	5	+
16	GEN-OFL-AUG-TET	1	-	-	-	4	+
17	CRX-AUG-CPR-TET	1	1	-	-	4	+
18	CAZ-CRX-CXM-OFL-CPR-AUG-TET	1	1	-	-	4	+
19	CAZ-CRX-AUG-TET	2	1	-	1	3	+
20	CAZ-CRX-CXM-AUG-TET	3	1	-	-	3	+
21	CRX-CXM-OFL-CPR-AUG-TET	1	-	-	-	4	+
22	CAZ-CRX-OFL-CPR-AUG-TET	1	-	-	-	4	+
23	CRX-CXM-GEN-AUG-CPR-TET	1	-	-	-	5	+
24	CAZ-CRX-CXM-AUG-CPR-TET	1	-	-	-	4	+
25	CAZ-CRX-CXM-GEN-OFL-CPR-AUG-TET	1	2	-	-	5	+
26	CAZ-CRX-GEN-OFL-AUG-TET	1	-	-	-	5	+
27	CRX-GEN-AUG-CPR-TET	-	1	1	-	5	+
28	OFL-AUG-CPR-TET	-	1	1	-	4	+
29	GEN-OFL-CPR-AUG-TET	-	-	1	1	4	+
30	CRX-GEN-AUG-TET	-	2	1	7	4	+
31	CAZ-GEN-AUG-TET	-	1	-	-	4	+
32	CAZ-GEN-OFL-CPR-AUG-TET	-	1	-	-	5	+
33	CAZ-CXM-GEN-OFL-CPR-AUG-TET	-	1	-	-	5	+
34	CAZ-CXM-OFL-CPR-AUG-TET	-	1	-	-	4	+
35	CXM-GEN-OFL-CPR-AUG-TET	-	1	-	-	5	+
36	CRX-GEN-OFL-CPR-AUG-NIT-TET	-	1	-	-	6	+
37	CAZ-CRX-CXM-GEN-OFL-AUG-TET	-	1	-	-	5	+
38	GEN-AUG-CPR-TET	-	1	-	-	4	+
39	CRX-CXM-AUG-TET	-	2	-	-	4	+
40	CAZ-CRX-CXM-OFL-AUG-TET	-	1	-	-	4	+
41	CAZ-CRX-GEN-AUG-TET	-	1	-	2	4	+
42	CRX-GEN-OFL-CPR-AUG-TET	-	-	-	4	5	+
43	CRX-GEN-OFL-AUG-TET	-	-	-	1	5	+

AUG: amoxicillin/clavulanic acid or Augmentin; CAZ: ceftazidime; CPR: ciprofloxacin; CRX: cefuroxime; CXM: cefixime; GEN: gentamicin; NIT: nitrofurantoin; OFL: ofloxacin; TET: tetracycline; MDR: multidrug-resistant

**Figure 2:**
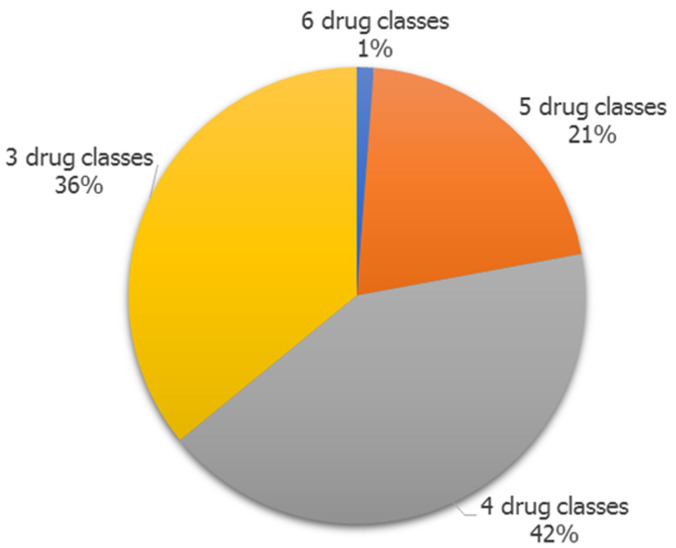
distribution of multidrug-resistant *E. coli* to different drug classes

**Figure 3:**
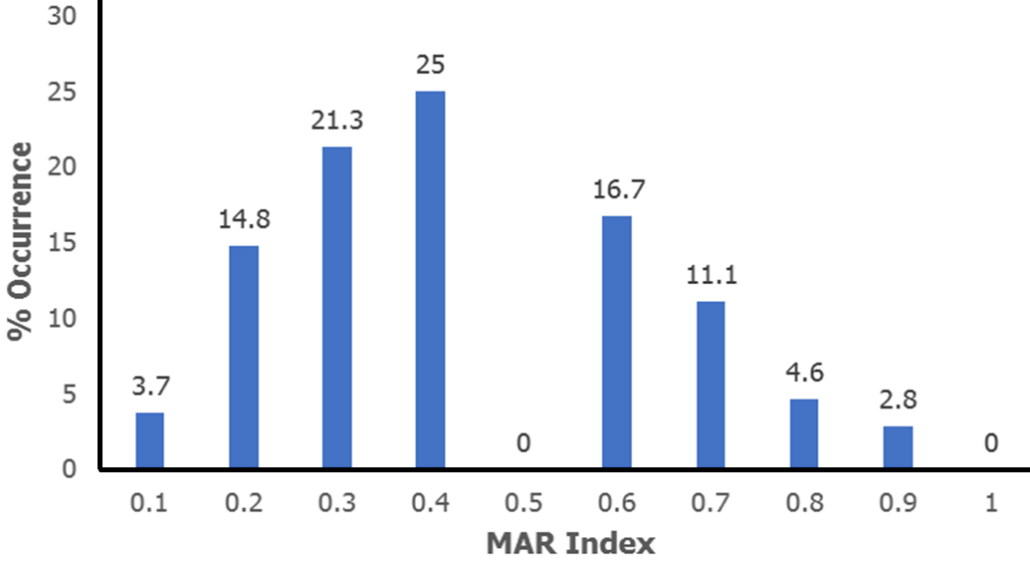
multiple antibiotic resistant index for *E. coli* isolates

The majority of isolates of *Escherichia coli* are resistant to three or more drug classes. Of the 108 isolates in this study, 83 (76.9%) are multidrug resistant (MDR). One isolate (1%) had the lowest resistance to six classes of antibiotics, while the highest resistance to four drug classes was 35 (42%) (Figure 2). Eighty-one and a half percent (81.5%) of isolates in the current study had MAR index values higher than 0.2. In this investigation, extreme MAR index values ≥ 0.80 were found (Figure 3).

**Figure 4:**
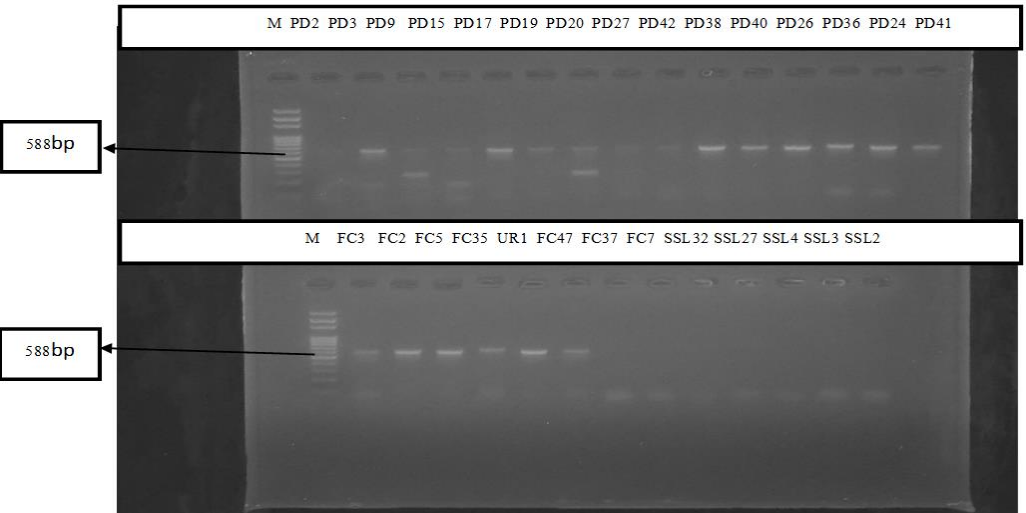
agarose gel electrophoresis of amplified EC16 gene products (588bp) from representative *Escherichia coli* isolates using a 100bp ladder

**Figure 5:**
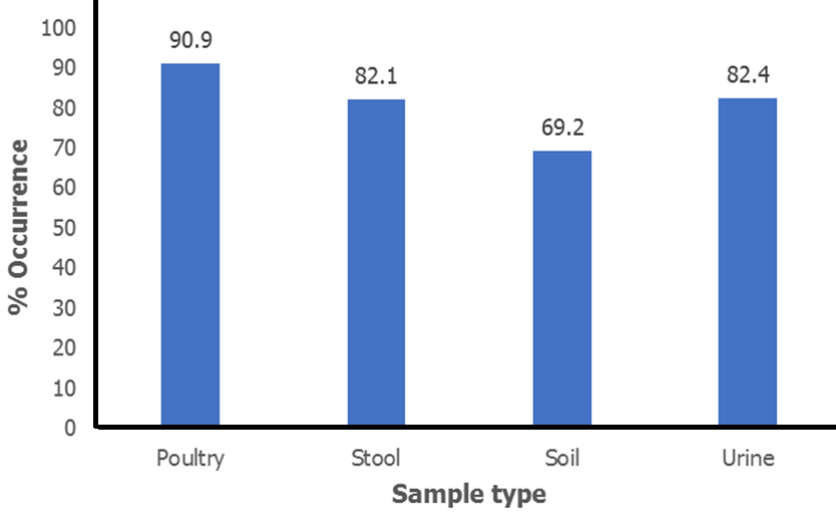
percentage of molecular confirmed *E. coli* isolates

Using the amplified 16S rRNA gene for *E. coli* isolates, the molecular presence of *Escherichia coli* was verified (Figure 4), and found that 82.9% (29 of 35) of the non-clinical isolates and 82.2% (60 of 73) of the clinical isolates were *E. coli*. Based on sample types, a breakdown of genotypic test confirmed *E. coli* reveals in urine 28/34 (82.4%), stool 32/39 (82.1%), soil 9/13 (69.2%), and poultry 20/22 (90.9%) were the most common sources of the bacteria (Figure 5).

## Discussion

This study aimed to identify the multiple antibiotic resistance (MAR) indices and molecularly identify *Escherichia coli* isolated from clinical and nonclinical sources in Port Harcourt Metropolis, Nigeria, in order to gain a better understanding of the prevalence and characteristics of antibiotic-resistant *E. coli* in the area. According to our findings, a significant percentage of *E. coli* isolates from both clinical and nonclinical sources exhibited resistance to various antibiotics, and the isolates had high MAR indices. The presence of *E. coli* bacteria with antibiotic resistance was detected, highlighting the possible public health consequences of antibiotic resistance in the region. Our study's noteworthy identification of a significant number of multidrug-resistant isolates and exceptionally high MAR index values of 0.8 and 0.9 *E. coli* isolates suggested the spread of various resistance genes in *E. coli* strains in Port Harcourt Metropolis. These findings demonstrate the need for more monitoring and careful antibiotic use in the region.

The study investigation noted a prevalence of 73% clinical and 35% non-clinical *E. coli* isolates biochemically. The current study's evaluation of resistant *E. coli* indicates that the high resistance to tetracycline, amoxicillin/clavulanic acid, and cefuroxime poses a significant risk of antibiotic-resistant *E. coli* in both clinical and non-clinical settings (Figure 1). This may be because antibiotics are still used in animal feed that is meant to stimulate growth rather than cure illnesses, and patients continue to abuse antibiotics without a doctor's prescription. The current study examined the antibiotic profile of *E. coli* and found 43 profiles among *E. coli* strains, which is comparable to studies by Ajuga *et al*. (2021), who discovered 51 antibiotic profiles or antibiograms among 61.4% of *E. coli* isolates [17]. According to research by Kallau *et al*. (2018), MDR *E. coli* was common and showed 39 different profiles in 57.3% of pig isolates [18]. Misuse of antibiotics leads to a rise in the profile of antibiotic resistance, which calls for monitoring and remedial measures.

*E. coli* strains that are resistant to three or more antibiotic classes are known as multidrug-resistant (MDR) strains. The current investigation shows that 1% of isolates are resistant to 6 antibiotic drug classes, 21% to 5 drug classes, 42% to 4 drug classes, and 36% to 3 drug classes. The rates found in this study are similar to those found by Pormohammad *et al*. (2019), who found that the overall MDR prevalence rate in isolates of *E. coli* from humans, the environment, and animals was 22%, 31.3%, and 5.7%, respectively [19]. According to reports by Suarez-Perez *et al*. (2021), the MDR prevalence of *E. coli* in chickens and other birds was 39.8% [20]. Furthermore, 85% of *E. coli* isolates from poultry had multidrug resistance in more than two drug classes, according to Amer *et al*. (2018) [21]. Together with the MDR data mentioned above, the study also found that the most common MAR index in *E. coli* isolates was 0.4 (= 0.4), which had a 25% prevalence. The extreme MAR index values of 0.8 and 0.9, respectively, were linked to 4.6% and 2.8% of isolates in this study. Similar conclusions that *E. coli* isolates had exceptionally high MAR index values and were reported in papers by Cookey *et al*. (2016), Otokunefor *et al*. (2018), Ajuga *et al*. (2021), and Datok *et al*. (2021) [1,13,17,22]. The choice of antibiotics for infection prevention and treatment is influenced by the MAR index results. When selecting antibiotics for therapeutic purposes, monitoring and testing for antibiotic susceptibility are essential because these MAR indices indicate that *E. coli* isolates have higher rates of resistance. Given the rising incidence of multidrug resistance in *E. coli*, the need for antibiotic surveillance programs in public health is more necessary.

EC16 primers were used in this study to molecularly identify 82.2% of clinical (60 of 73) and 82.9% of non-clinical *Escherichia coli*, showing a high degree of agreement between phenotypic and genotypic testing methods. 90.9% of *E. coli* was detected in poultry, 82.1% in stool, 69.2% in soil, and 82.4% in urine, according to the study. Genomic *E. coli* confirmation across the tested sample types showed a higher prevalence of these organisms in poultry (90.9%) [23], which is comparable to studies by Moawad *et al*. (2018) that show a high frequency of *E. coli* in poultry with 87.5% (63 of 72 samples). Ajuga *et al*. (2021) reported moderate rates of 62.7% (52 of 83) from non-clinical isolates of *E. coli* [17]. Increased surveillance for infection control is indicated by the high genotypic prevalence of *E. coli* and the rate of multiple antibiotic resistance in *E. coli* isolated from clinical and environmental sources. Developing reliable antibiotic strategies and tracking and evaluating antibiotic-sensitivity trends may lead to better outcomes for the inhibition and control of *E. coli* infections.

The studies used molecular methods to detect and describe antibiotic-resistant *E. coli* bacteria, provides a complete understanding of the antibiotic resistance profiles in the region. Nevertheless, the sampling area was limited to particular clinical (urine and stool) and nonclinical sources (soil and poultry droppings), which might not be representative of the entire region, and the study's sample size was small. The study highlights the necessity for further surveillance and research in this field and contributes to the growing body of information regarding antibiotic resistance in Nigeria. The results can direct the creation of focused interventions to halt the spread of antibiotic-resistant *E. coli* in Port Harcourt Metropolis, and the approach can be adjusted for use in comparable research in other areas.

## Conclusion

A high risk of resistant *E. coli* in the samples being analyzed is suggested by the current study's significant level of antibiotic resistance in *E. coli*. The level of MDR discovered in this investigation might indicate antibiotic overuse. Elevated MAR index values indicate the failure of antibiotic treatment and identify potential sources of antibiotic resistance genes. The potential for human transmission of MDR *E. coli* strains is highlighted by the strains' presence in the environmental samples that were examined. It is recommended that strict guidelines be implemented to restrict the use of antibiotics in human and animal feed in order to prevent the emergence of numerous antibiotic-resistant strains that are circulating in the environment. The study's findings show an increase in the prevalence of antibiotic-resistant *E. coli* in this area.

### What is known about this topic

- The resistance of E. coli to commonly administered antibiotics is a typical occurrence;
- MAR index is used to identify sources of potential risk of antibiotic-resistant organisms;
- High levels of MDR are an indicator of antibiotics misuse.

### What this study adds

- The study revealed 76.9% MDR E. coli isolates, with 42% resistance to 4 drug classes and 1% to 6 antibiotic classes;
- Twenty-five percent of isolates had a MAR-index of 0.4, while 4.6% and 2.8% had high MAR-index values of 0.8 and 0.9, respectively;
- The genotypic test confirmed 82.2 % clinical and 82.9% non-clinical E. coli isolated from Port Harcourt, Nigeria.
